# Transcriptional Response in Mouse Thyroid Tissue after ^211^At Administration: Effects of Absorbed Dose, Initial Dose-Rate and Time after Administration

**DOI:** 10.1371/journal.pone.0131686

**Published:** 2015-07-15

**Authors:** Nils Rudqvist, Johan Spetz, Emil Schüler, Toshima Z. Parris, Britta Langen, Khalil Helou, Eva Forssell-Aronsson

**Affiliations:** 1 Department of Radiation Physics, Institute of Clinical Sciences, Sahlgrenska Cancer Center, Sahlgrenska Academy, University of Gothenburg, Gothenburg, Sweden; 2 Department of Oncology, Institute of Clinical Sciences, Sahlgrenska Cancer Center, Sahlgrenska Academy, University of Gothenburg, Gothenburg, Sweden; University of California Davis, UNITED STATES

## Abstract

**Background:**

^211^At-labeled radiopharmaceuticals are potentially useful for tumor therapy. However, a limitation has been the preferential accumulation of released ^211^At in the thyroid gland, which is a critical organ for such therapy. The aim of this study was to determine the effect of absorbed dose, dose-rate, and time after ^211^At exposure on genome-wide transcriptional expression in mouse thyroid gland.

**Methods:**

BALB/c mice were i.v. injected with 1.7, 7.5 or 100 kBq ^211^At. Animals injected with 1.7 kBq were killed after 1, 6, or 168 h with mean thyroid absorbed doses of 0.023, 0.32, and 1.8 Gy, respectively. Animals injected with 7.5 and 100 kBq were killed after 6 and 1 h, respectively; mean thyroid absorbed dose was 1.4 Gy. Total RNA was extracted from pooled thyroids and the Illumina RNA microarray platform was used to determine mRNA levels. Differentially expressed transcripts and enriched GO terms were determined with adjusted p-value <0.01 and fold change >1.5, and p-value <0.05, respectively.

**Results:**

In total, 1232 differentially expressed transcripts were detected after ^211^At administration, demonstrating a profound effect on gene regulation. The number of regulated transcripts increased with higher initial dose-rate/absorbed dose at 1 or 6 h. However, the number of regulated transcripts decreased with mean absorbed dose/time after 1.7 kBq ^211^At administration. Furthermore, similar regulation profiles were seen for groups administered 1.7 kBq. Interestingly, few previously proposed radiation responsive genes were detected in the present study. Regulation of immunological processes were prevalent at 1, 6, and 168 h after 1.7 kBq administration (0.023, 0.32, 1.8 Gy).

## Introduction

Cellular responses to stimuli can be studied on different molecular levels, including gene expression regulation. RNA microarray analysis is a high-throughput semi-quantitative technique that enables measurement of genome-wide transcriptional gene regulation. By comparing genome-wide transcriptional levels between samples, e.g. irradiated and non-irradiated thyroids, a differential gene expression profile can be generated illustrating gene up- and downregulation for each tissue sample. This technique allows for the assessment of changes in cellular activity due to radiation exposure and facilitates the identification of radiation biomarkers [1].

^211^At-labeled radiopharmaceuticals are potentially useful for tumor therapy [2, 3]. However, due to chemical similarities with iodide, free ^211^At accumulates in the thyroid gland [4, 5]. During metabolism of ^211^At-labeled radiopharmaceuticals, free ^211^At may be released, resulting in significant thyroid gland exposure [6]. Therefore, the thyroid gland is one of the critical organs during treatment with ^211^At-labeled radiopharmaceuticals. ^211^At emits alpha particles, and compared with the beta particles emitted from the more routinely used ^131^I, alpha particles emitted from ^211^At have approximately a 200 times higher LET value and will deposit energy over a shorter range [7–9]. To deposit an average absorbed dose of 1 Gy to the follicular cell nucleus, 800 beta particle tracks are needed from ^131^I exposure, but only three alpha particle tracks from ^211^At exposure [10]. Additionally, ^211^At has a half-life of 7 hours compared with 8 days for ^131^I. Altogether, this results in a different exposure setting for ^211^At compared with ^131^I.

We have conducted several *in vivo* studies using RNA microarray technology to evaluate transcriptional responses in mouse thyroid and non-thyroidal tissues after ^131^I and ^211^At administration, as well as mouse kidney after ^177^LuCl_3_ and ^177^Lu-octreotate administration [11–16]. Microarray analysis of mouse thyroid tissue 24 h after various amounts of ^211^At (mean absorbed dose to thyroid of 0.05–32 Gy) demonstrated a complex dose response pattern, with more transcripts regulated at lower absorbed doses (0.05 and 0.5 Gy) [11]. To continue in this research field, it is also of great interest to study effects of dose-rate and time after administration.

The aim of this work was to investigate radiation-induced effects of on thyroid tissue in mice after ^211^At administration using RNA microarray technique. The intention was to determine transcriptional variations due to dose-rate and time after exposure. The transcriptional response to injection of 1.7 kBq ^211^At was investigated at 1, 6, and 168 h (0.023, 0.32, and 1.8 Gy to thyroid) to gain further knowledge of the effects of a similar initial dose-rate. The amount of 1.7 kBq ^211^At was chosen since we have previously investigated the transcriptional response to 1.7 kBq ^211^At, but at 24 h (1.4 Gy to thyroid). In a similar manner, the transcriptional response to an absorbed dose of 1.4 Gy at 1 and 6 h (100 and 7.5 kBq injected activity, respectively) was investigated to gain knowledge of the effect of a similar absorbed dose but at different time-points after injection.

## Materials and Methods

### Radionuclide and radioactivity measurements

^211^At (t_½_: 7.2 h) was produced at the Cyclotron and PET Unit at Rigshospitalet in Copenhagen, Denmark, using the ^209^Bi(α,2n)^211^At reaction, and free ^211^At was prepared as described previously [17]. ^211^At was produced using 28 MeV α-particles, and the ratio between produced activity of ^210^At and ^211^At has been determined to 2.5*10^−8^. ^211^At was introduced in water solution as late as possible, just prior to injection, in order to assure that most of ^211^At was kept as astatide.

A Wallac 1480 Wizard 3" gamma counter (Wallac Oy, Turku, Finland) was used to determine ^211^At activity in stock solutions. The gamma counter was calibrated and routinely checked for clinical use, and to determine ^211^At activity, the energy window was positioned to include only characteristic x-rays emitted from ^211^Po with energies between 72 and 88 keV. Correction was done for background and dead-time losses, although the dead-time factor was generally less than 1.005.

### Dosimetric calculations

Calculation of absorbed dose from alpha particles emitted by ^211^At and its daughter nuclide ^211^Po located in the thyroid using the Medical Internal Radiation Dose (MIRD) formalism has been previously described [11, 18]. ^211^At decays to either ^211^Po (58%) by electron capture (EC) or to ^207^Bi (42%) by emission of mainly 5.87 MeV α-particles [19]. ^211^Po then quickly (t_½_: 0.52 s) decays to ^207^Pb (stable) with emission of mainly 7.45 MeV α-particles, while ^207^Bi decays much slower (t_½_: 31.5 y) by EC to ^207^Pb. Almost 99% of all energy emitted by ^211^At is α-particles (including the decay branches to stable ^207^Pb). The mean absorbed dose to thyroid was determined using mean alpha particle energy per decay for emitted alpha particles from ^211^At and ^211^Po (2.50 and 7.58 MeV, respectively) and the ^211^At and ^211^Po was assumed homogeneously distributed in the thyroid. Previously published data on activity concentrations in mouse thyroid tissue was used to determine time-integrated activity between 1 and 24 h [20]. The absorbed dose delivered from administration to 168 h after administration of ^211^At was estimated presuming no change in activity concentration between 24 and 168 h (except for physical decay). A standard thyroid mass of 3 mg and an absorbed fraction of 1 were used. In the present study, initial mean dose-rate was defined as the mean absorbed dose deposited during the first hour after administration of ^211^At.

### Study design

The study design was approved by the Ethical Committee on Animal Experiments in Gothenburg, Sweden. Eighteen female BALB/c nude mice (CAnN.Cg-Foxn1nu/Crl, Charles River Laboratories International, Inc., Salzfeld, Germany) were divided into six groups (n = 3). Exposure parameters are summarized in Table 1. Animals in three groups were intravenously (i.v.) injected with 1.7 kBq ^211^At and killed 1, 6, and 168 h after administration. Animals in two groups were i.v. injected with 100 and 7.5 kBq ^211^At and killed at 1 and 6 h after administration, respectively. ^211^At was administered in 0.15 mL phosphate-buffered saline (pH 7). Animals in the sixth group were mock-treated with a syringe in the tail-vein and used as controls. Water and standard laboratory mouse chow were given *ad libitum*. Before killing the animals with cardiac puncture, animals were anesthetised using pentobarbitalnatrium (APL; Kungens Kurva, Sweden). After killing the animals, the thyroids were quickly excised and snap-frozen in liquid nitrogen. Thyroids were removed surgically by a skilled animal technician using autoclaved surgical scissors, scalpels, and tweezers. Images of hematoxylin-eosin stained microtome sections of female BALB/c nude mouse thyroid tissue removed using the same technique have been published elsewhere [21].

**Table 1:** Exposure parameters and number of regulated transcripts.

				No. of regulated transcripts		
Exposure time (h)	Injected activity (kBq)	Mean absorbed dose (Gy)	Initial mean dose-rate (Gy/h)	Total	Down	Up
1	1.7	0.023	0.023	322	198	124
1	100	1.4	1.4	851	401	450
6	1.7	0.32	0.023	227	160	67
6	7.5	1.4	0.10	424	256	168
168	1.7	1.8	0.023	185	129	56

Exposure time indicates the time animals were alive after i.v. injection of ^211^At. Initial mean dose-rate is defined as the absorbed dose deposited during the first hour after ^211^At administration.

### Transcriptional analysis

Total RNA extraction and the RNA microarray technique have been described elsewhere [11]. Briefly, we extracted total RNA from pooled thyroids, i.e. thyroids from animals within each group. The RNA samples were processed in triplicate at the Swegene Center for Integrative Biology at Lund University (SCIBLU) using the MouseRef-8 Whole-Genome Expression BeadChips (Illumina; San Diego, CA, USA). Raw signal intensities were acquired, and preprocessing and normalization of the data was performed using the BioArray Software Environment system (BASE) [22]. Differentially expressed (herein synonymous to *regulated*) transcripts (fold change > 1.5, Benjamini-Hochberg False Discovery Rate corrected p-value < 0.01) were determined using Nexus Expression 3.0 (BioDiscovery; El Segundo, CA). Raw data have been deposited at NCBI Gene Expression Omnibus (GEO no. GSE66089).

The regulated transcripts identified after ^211^At exposure were compared with 102 previously proposed radiation biomarkers [23–26].

Hierarchical clustering was performed using the hclust function (stats package, version 3.1.1) with the complete linkage algorithm and Lance-Williams dissimilarity update formula. Heat maps were created with the heatmap.2 function (gplots package, version 2.14.2). Both functions were used in the R statistical computing environment (http://www.r-project.org) [27].

Regulated transcripts associated with biological processes were characterized using Gene Ontology (GO) terms (Nexus Expression 3.0, p-value <0.05). GO terms were further divided into eight main categories (with corresponding subcategories): 1) DNA integrity, 2) gene expression integrity, 3) cell communication, 4) organismic regulation, 5) cell cycle and differentiation, 6) metabolic processes, 7) cellular integrity, and 8) stress responses.

## Results

### Dosimetry

Exposure parameters are shown in Table 1. The mean absorbed dose to the thyroid tissue was calculated to 0.023 and 1.4 Gy at 1 h, 0.32 and 1.4 Gy at 6 h, and 1.8 Gy at 168 h after administration of ^211^At (Table 1). Injection of 1.7, 7.5 and 100 kBq resulted in an initial dose-rate to thyroid of 0.023, 0.1 and 1.4 Gy/h, respectively.

### Regulated transcripts

**Fig 1:**
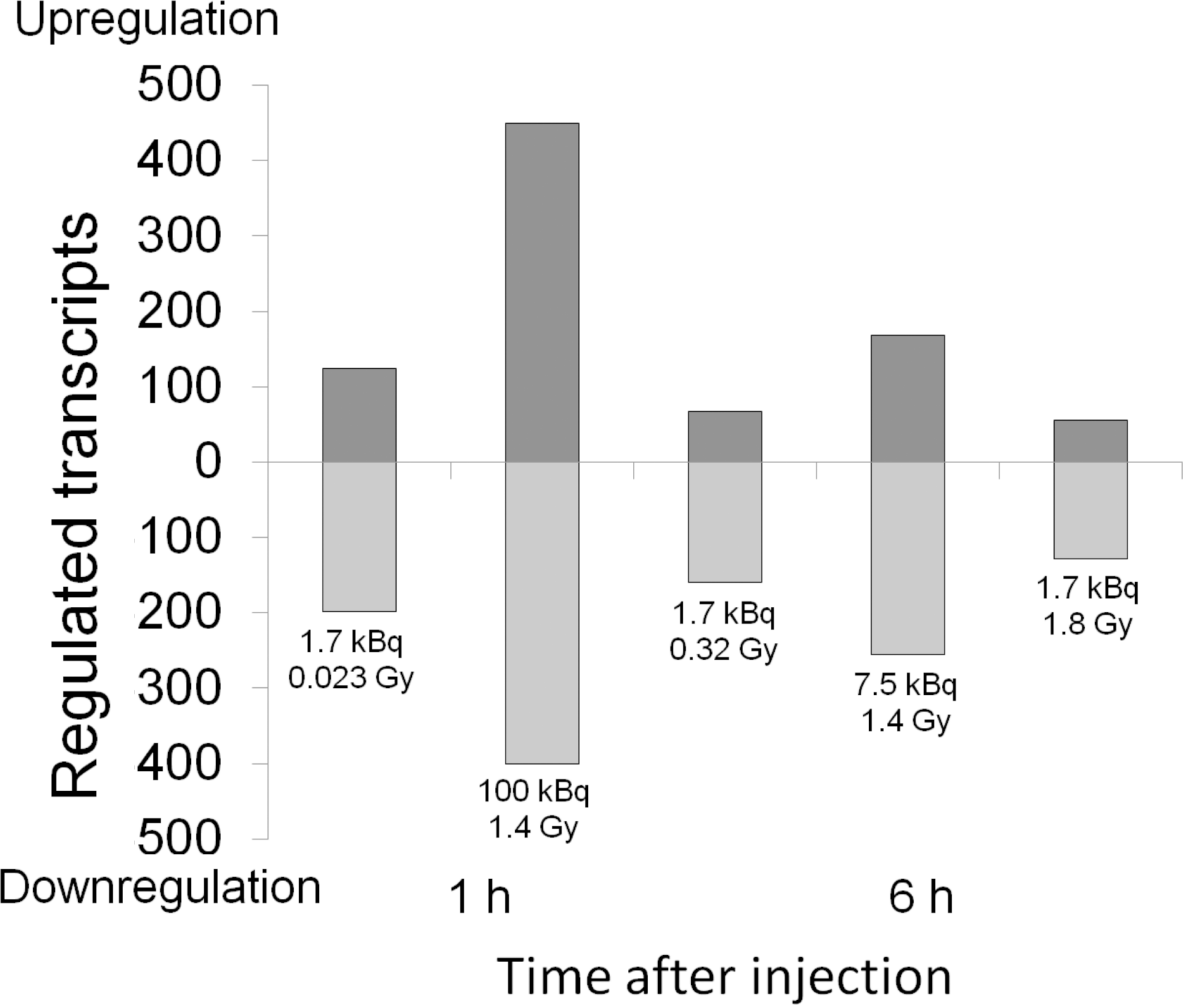
Number of regulated transcripts. Number of regulated transcripts (fold change > 1.5, adjusted p-value < 0.01) in mouse thyroid tissue 1–168 h after 1.7–100 kBq ^211^At administration.

In total, 1232 regulated transcripts were identified. The number of regulated transcripts in each group varied between 185 (168 h, 1.7 kBq, 1.8 Gy) and 851 (1 h, 100 kBq, 1.4 Gy), where downregulation was most prevalent (Fig 1). The number of regulated transcripts decreased with time and absorbed dose after 1.7 kBq ^211^At administrations, but increased with initial dose-rate for the same absorbed dose (1.4 Gy) when comparing 1 h with 6 h. Furthermore, the number of regulated transcripts increased with initial dose-rate/absorbed dose at both 1 and 6 h after administration.

**Fig 2:**
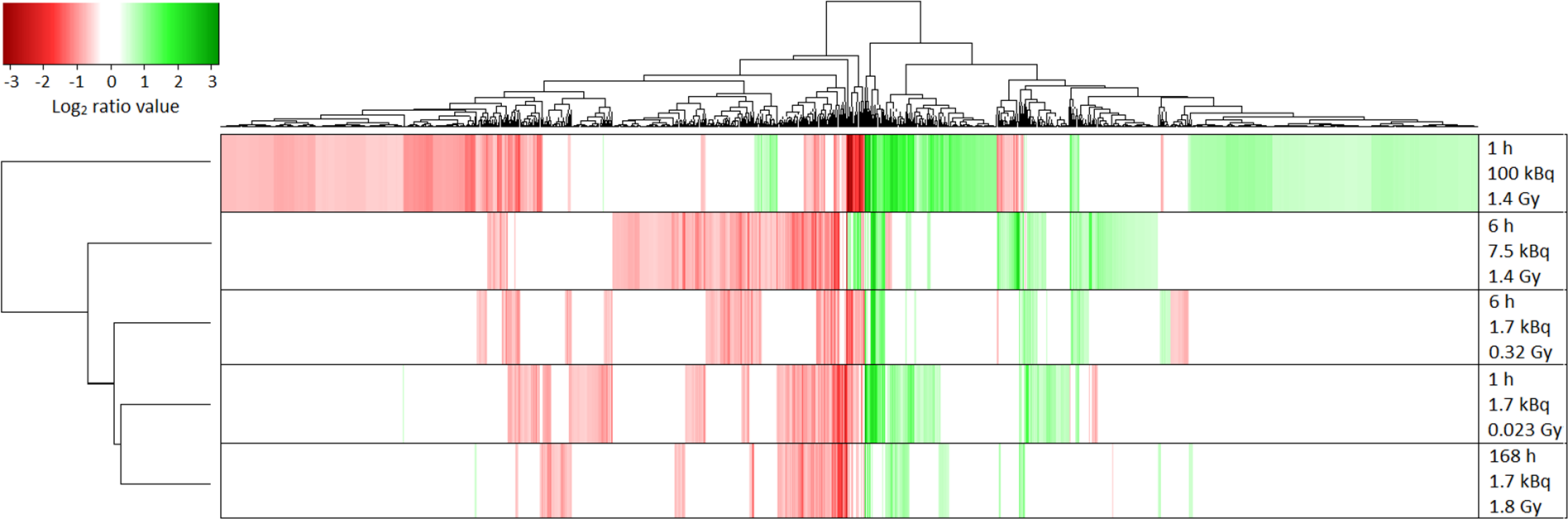
Heat map and hierarchical clustering of all 1232 regulated genes. Hierarchical clustering was performed using the hclust function with the complete linkage algorithm and Lance-Williams dissimilarity update formula in the R statistical computing environment (http://www.r-project.org). Heat maps were created with the heatmap.2 function within the gplots package.

There were substantial differences between the regulation profiles for different exposures (Fig 2). Hierarchical clustering revealed similarities between regulation profiles for animals administered 1.7 kBq and killed at 1, 6, and 168 h (0.023, 0.32, and 1.4 Gy). The regulation profiles for the remaining two groups were more distinct, in particular for animals 1 h after receiving 100 kBq ^211^At.

### Genes specific for certain exposure conditions

Genes specific for the following exposure conditions are shown in Table 2: 1 h or 6 h, 1.4 Gy, or 1.7 kBq ^211^At (fold change for these genes is given in S1 Table). At 1 or 6 h after administration, 42 and 43 genes, respectively, were exclusively regulated. At 1 h, upregulation was as common as downregulation, but at 6 h, downregulation was more prominent. Additionally, genes exclusively regulated 1 h and 6 h after ^211^At administration were generally regulated in the same direction, independent of dose-rate/absorbed dose. After 1.4 Gy (at 1 h and 6 h after ^211^At administration), 43 genes were exclusively regulated. Seventeen of these 43 genes were downregulated at 1 h but upregulated at 6 h, and 13 were upregulated at 1 h and downregulated at 6 h, i.e. their regulation was dependent on time after administration/initial dose-rate. Only 1 gene (*Casp1*) was exclusively regulated 1, 6, and 168 h after 1.7 kBq administration, while 9, 9 and 4 genes were exclusively regulated (in the same direction) at 1 h and 6 h, 1 h and 168 h, and 6 h and 168 h, respectively.

**Table 2:** Exposure-specific regulated genes in thyroid.

**Genes only regulated at 1 h (absorbed dose: 0.023 Gy or 1.4 Gy)**		
↑↑	[21]	*2310042D19Rik*, *8430408G22Rik*, *Acadm*, *Acot4*, *Asah3l*, *BC049806*, *Cnnm2*, *Cpt1b*, *Gm129*, *Gmpr*, *Hspb1*, *Kcnk3*, *LOC100047934*, *Lpin1*, *Pank3*, *Pck1*, *Rbp7*, *Sephs2*, *Slc25a20*, *Rbp7*, *Sephs2*, *Slc25a20*, *Slc27a2*, *Snrk*
↓↓	[19]	*Aif1*, *Arg1*, *Arhgap29*, *B9d1*, *Cd27*, *Csprs*, *D0H4S114*, *D14Ertd668e*, *Gna14*, *H1f0*, *H3f3b*, *Hist2h3c1*, *Hmgn2*, *Hspb6*, *Kndc1*, *LOC100043257*, *Pik3cg*, *Pml*, *Slamf9*
↑↓	[1]	*Iqgap2*
↓↑	[1]	*Gbp3*
**Genes only regulated at 6 h (absorbed dose: 0.32 Gy or 1.4 Gy)**		
↑↑	[9]	*4833439L19Rik*, *Bcl6*, *Def6*, *Nr1d2*, *Rhou*, *Rorc*, *Slc46a3*, *Smpdl3b*, *Tcfap2c*
↓↓	[34]	*1810030N24Rik*, *Acat2*, *Acsm3*, *Acss2*, *Akr1c12*, *Amy1*, *Arl4a*, *Atg10*, *Ccdc80*, *Elovl5*, *Elovl6*, *Emcn*, *Enpp2*, *Gyg*, *Hrsp12*, *Insig1*, *Lyrm5*, *Mbnl1*, *Mcts1*, *Lyrm5*, *Mbnl1*, *Mcts1*, *Nsdhl*, *Obfc2a*, *Ppp1r3c*, *Psmb7*, *Ptger3*, *Rasl12*, *Rpl30*, *Sh3bgrl*, *Snn*, *Sspn*,*Vldlr*, *Vps35*, *Zeb2*
**Genes only regulated after 1.4 Gy exposure (exposure time: 1h and 6 h)**		
↓↓	[13]	*Cldn10*, *Elf5*, *Fgf10*, *Ivns1abp*, *Laptm4a*, *Lmo2*, *Maea*, *Nupr1*, *P2rx4*, *Pdcd4*, *Ppp1cb*, *Smpdl3a*, *Spon2*
↑↓	[13]	*Adhfe1*, *Agpat2*, *AU018778*, *Dgat2*, *Gja1*, *LOC333331*, *Maob*, *Mtch2*, *Pmp22*, *Pygl*, *Sbk*, *Slc25a1*, *Sorbs1*
↓↑	[17]	*Actn3*, *Ampd1*, *Car12*, *Csrp3*, *Eef1a2*, *Hrc*, *Klk1b11*, *Muc13*, *Mybpc2*, *Myh2*, *Myh4*, *Myoz1*, *Pfkm*, *Sidt1*, *Tcap*, *Ttn*, *Wfdc2*
**Genes only regulated after 1.7 kBq administration (exposure time: 1 h, 6 h, and 168 h)**		
↑↑ -	[1]	*Ifi30*
↓↓ -	[8]	*Gbp1*, *Gbp2*, *Gsta3*, *Ifit3*, *OTTMUSG00000000971*, *Samd9l*, *Tpmt*, *Usp18*
- ↑↑	[1]	*Oas1g*
- ↓↓	[3]	*2310057J18Rik*, *Fhl1*, *OTTMUSG00000008911*
↓ - ↓	[9]	*C1qb*, *C1qc*, *C4b*, *Cfp*, *Col6a1*, *Ear11*, *Ly6a*, *Lyzs*, *Mup2*, *Psp*
↓↓↓	[1]	*Casp1*

Exposure-specific regulated genes in thyroid shared between two or three exposure conditions (and unique for that condition) at similar exposure time (1h or 6h), at similar absorbed dose (1.4 Gy) or at similar injected activity (1.7 kBq). Arrows indicate direction of regulation for exposure conditions defined within parentheses. ↑ indicates upregulation and ↓ indicates downregulation of genes. The number of genes are given in brackets. For example, at 1 h after ^211^At administration the *2310042D19Rik* gene was exclusively upregulated at both 0.023 and 1.4 Gy. The absorbed doses given are mean absorbed doses to the thyroid.

### Recurrently regulated genes

**Fig 3:**
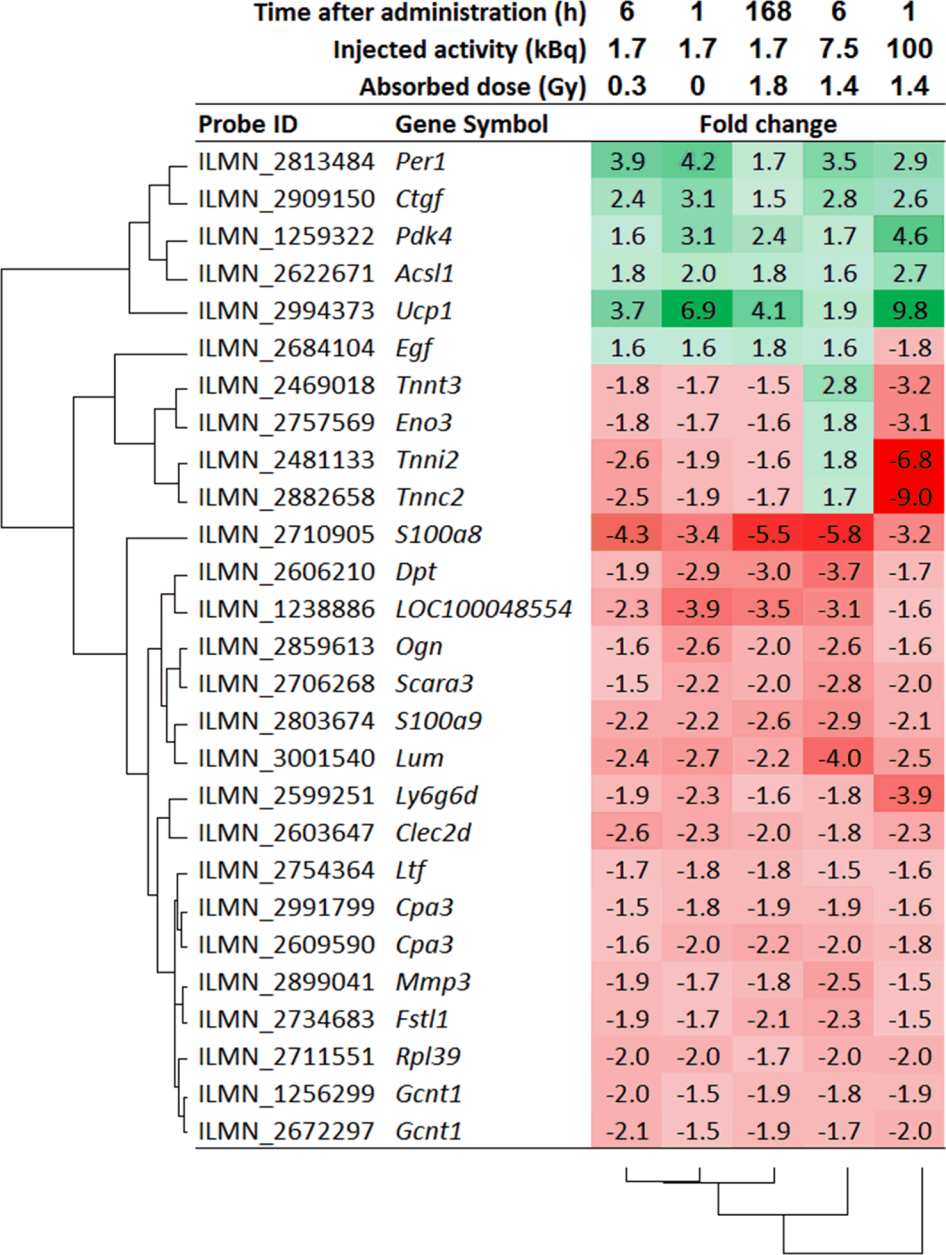
Recurrently regulated genes. Genes regulated in all irradiated groups after ^211^At administration. Hierarchical clustering was performed using the hclust function with the complete linkage algorithm and Lance-Williams dissimilarity update formula in the R statistical computing environment (http://www.r-project.org) [27]. Red and green color indicate down- and upregulation, respectively.

Twenty-five genes were recurrently regulated at all exposures (Fig 3). Using these 25 genes, hierarchical clustering of the irradiated groups was similar to that when including all 1232 genes (Figs 2 and 3). Animals administered 1.7 kBq and killed at 1, 6, and 168 h (0.023, 0.32, 1.8 Gy) were clustered together. The regulation profiles for animals receiving 7.5 and 100 kBq clustered alone, although the 7.5 kBq regulation profile more closely resembled that of the remaining groups compared with the 100 kBq regulation profile. Three large clusters were identified among the 25 recurrently regulated genes: i) upregulation (6 genes), ii) mixed direction of regulation (5 genes), and iii) downregulation (15 genes). Among the genes with mixed direction of regulation, 4 genes were upregulated 6 h after 7.5 kBq administration but downregulated in remaining groups, and 1 gene was downregulated 1 h after 100 kBq administration but upregulated in the remaining groups.

#### Previously proposed genes associated with radiation

Of 102 genes previously associated with radiation, only 11 genes were regulated in the present study: *Amy1*, *Ccnd1*, *Ccng1*, *Cdkn1a*, *Gadd45g*, *Gja1*, *Gjb2*, *Hspe1*, *Lep*, *Tgfbr2*, and *Tnfrsf21* (Table 3) [23–26]. None of the previously radiation-associated genes were regulated in all groups, although the *Gadd45g* gene was recurrently regulated in four groups: 1 h and 6 h after ^211^At administration.

**Table 3:** Validation of 102 previously proposed radiation-related genes.

Time after administration (h)		1	1	6	6	168
Injected activity (kBq)		1.7	100	1.7	7.5	1.7
Absorbed dose (Gy)		0.023	1.4	0.32	1.4	1.8
Probe ID	Gene Symbol	Fold change				
ILMN_2626453	*Amy1*			-1.8	-1.7	
ILMN_2601471	*Ccnd1*		-1.5			
ILMN_2500276	*Ccng1*			-1.8		
ILMN_2710229	*Ccng1*				-1.5	
ILMN_2634083	*Cdkn1a*	2.3	3.0		1.7	
ILMN_2744890	*Gadd45g*	1.9	2.0	1.8	1.6	
ILMN_2903945	*Gadd45g*	2.3	2.3	2.2	2.0	
ILMN_1244291	*Gja1*		1.9		-1.5	
ILMN_1227148	*Gjb2*	1.5			1.5	
ILMN_2999627	*Gjb2*	1.6			1.7	
ILMN_2960308	*Hspe1*		1.6			
ILMN_2695964	*Lep*		1.7	-1.8	-2.1	
ILMN_2760979	*Tgfbr2*	-1.6			-2.2	
ILMN_2901626	*Tnfrsf21*	1.5			1.7	

Remaining previously proposed radiation-related genes were not regulated in the present study.

### Gene Ontology (GO) terms

Distinct differences in affected biological processes were identified in the present study (Table 4). An impact on immune response (main category: stress response) was more pronounced 1, 6, and 168 h after 1.7 kBq administration, where effects on complement activation, defense response, and general and innate immune response were identified at 1 h and 168 h after 1.7 kBq administration. However, at 6 h after 1.7 kBq administration the response involved positive regulation of several interleukins and protection from NK cell mediated cytotoxicity. Additionally, GO terms related to inflammation were only identified 168 h after 1.7 kBq administration. For cellular integrity, more GO terms were identified at 6 h compared with 1 h and 168 h, although different subcategories were affected at this time point: predominantly cytoskeleton & motility after 1.7 kBq and extracellular matrix & cellular membrane after 7.5 kBq. Ontogenesis and systemic regulation (main category: organismic regulation) was affected according to the identified GO terms. For ontogenesis, the highest impact was detected 6 h after 7.5 kBq administration, followed by 168 h after 1.7 kBq, while a similar number of GO terms related to systemic regulation were found for all groups. The most prominent impact on metabolism was identified in the group with the highest initial dose-rate.

**Table 4:** Categorized Gene Ontology terms.

* *Time after administration (h)	1	1	6	6	168
* *Injected activity (kBq)	1.7	100	1.7	7.5	1.7
* *Mean absorbed dose (Gy)	0.023	1.4	0.32	1.4	1.8
* *Initial mean dose-rate (Gy/h)	0.023	1.4	0.023	0.10	0.023
***Cell communication***	***2***	***2***	***4***	***3***	***1***
Intercellular signaling	0	0	2	1	0
Signal transduction	2	2	2	2	1
***Cell cycle and differentiation***	***1***	***3***	***3***	***2***	***5***
Apoptotic cell death	0	1	1	0	0
Cell cycle regulation	0	0	0	1	1
Cell death	1	0	0	0	1
Differentiation & aging	0	2	2	1	3
***Cellular integrity***	***3***	***5***	***8***	***7***	***4***
Cytoskeleton & motility	1	0	4	1	2
Extracellular matrix & cellular membrane	0	0	1	4	1
General	0	1	0	0	0
Physico-chemical environment	1	1	2	2	1
Supramolecular maintenance	1	3	1	0	0
***DNA integrity***	***2***	***2***	***2***	***0***	***0***
Chromatin organization	2	2	2	0	0
***Gene expression integrity***	***1***	***0***	***1***	***0***	***0***
Transcription	1	0	1	0	0
***Metabolism***	***7***	***19***	***8***	***7***	***9***
Carbohydrates	1	2	0	1	1
General	1	3	1	0	0
Lipids, fatty acids	2	5	1	2	3
Nucleic acid related	0	0	2	0	0
Other	2	5	2	1	4
Proteins, amino acids	1	2	2	3	1
Signaling molecules	0	2	0	0	0
***Organismic regulation***	***5***	***5***	***6***	***11***	***8***
Ontogenesis	2	1	1	8	4
Systemic regulation	3	4	5	3	4
***Stress responses***	***8***	***2***	***7***	***2***	***13***
Immune response	7	1	5	2	6
Inflammatory response	0	0	0	0	4
Other	1	1	2	0	3

Regulated transcripts were associated with biological functions using Gene Ontology (GO) terms (Nexus Expression 3.0, p-value < 0.05). GO terms were divided into eight main categories (with corresponding subcategories): 1) cell communication; 2) cell cycle and differentiation; 3) cellular integrity; 4) DNA integrity; 5) gene expression integrity; 6) metabolism; 7) organismic regulation; 8) stress responses. Initial mean dose-rate is defined as the absorbed dose deposited in the first hour after injection of ^211^At. The data given are the number of identified GO terms for each category and subcategory. The gray scale indicates the number of identified GO terms in a subcategory, i.e. white color indicates no identified GO terms and darkest gray color indicates 8 (max) GO terms.

## Discussion

The thyroid gland will most likely be exposed after administration of ^211^At-labeled pharmaceuticals. In the present study, transcriptional variations dependent on dose-rate, absorbed dose, and time after administration were investigated. When working with radionuclides, is it not possible to study each parameter individually while allowing the other parameters to remain constant. This is due to intrinsic dependencies between the parameters; changing one parameter inevitably results in changing others. Additionally, gene expression regulation is a dynamic process, where the biological response time is different for different genes and biological processes. Thus, the total response will both include effects of physical exposure parameters and differences in biological response times. To study the effects of dose-rate and time after administration, animals were injected with 7.5 and 100 kBq, and killed at 6 h and 1 h, respectively. Both of these exposures result in an absorbed dose of 1.4 Gy to the thyroid, allowing an investigation of the transcriptional response to the same absorbed dose but with different dose-rates and time after administration. To study transcriptional regulation at different time-points and absorbed doses from a similar treatment, animals were injected with 1.7 kBq and killed at 1, 6, and 168 h. In these animals, the dose-rate at a given time-point (and initial dose-rate) will be similar, but as time after injection increases, so will also the absorbed dose. To investigate the effect of variations in absorbed dose and dose-rate on the transcriptional response, animals were injected with various amounts of ^211^At and killed at the same time after administration. Altogether, the experimental setup in the present study allowed a comprehensive assessment of how variations in exposure parameters affect transcriptional regulation.

Many regulated transcripts were identified in mouse thyroid tissue in response to ^211^At exposure, including distinct differences in response between exposures. The number of regulated transcripts increased with absorbed dose/dose-rate at both 1 h and 6 h. Allowing the absorbed dose to remain constant at 1.4 Gy and killing the animals at 1 h and 6 h resulted in an increased number of regulated transcripts with dose-rate and a decreased number of regulated transcripts with time after administration. After administration of 1.7 kBq and killing animals after 1, 6, and 168 h, the number of regulated transcripts decreased with increased time after administration/absorbed dose. Additionally, regulation profiles for animal groups exposed to 1.7 kBq at 1, 6, and 168 h clustered together, with minimal similarities to animal groups exposed to 1.4 Gy (1 and 6 h). Taken together, these results suggest that the ^211^At-induced transcriptional response is more dependent on initial dose-rate compared with absorbed dose and time after injection.

We have previously studied the transcriptional response in thyroid tissue 24 h after ^131^I and ^211^At administrations [11, 13]. These studies showed that the number of regulated transcripts varied with absorbed dose/dose-rate in a similar way with the lowest number of regulated transcripts in the groups that received the highest absorbed dose/dose-rate, but the dose-response relationship was not fully monotonous. Differences between results from these studies and the present study may be due to temporal differences, and in the case of ^131^I, differences in radiation quality. For example, ^211^At mainly emits high LET alpha particles while ^131^I mainly emits low LET electrons; high LET particles deposit energy through direct interaction, while low LET particles deposit energy mainly through indirect action (i.e. by producing free radicals). Furthermore, differences in e.g. half-life (7.2 h and 8.0 d for ^211^At and ^131^I, respectively), range of emitted particles, and relative number of cells hit likely affects changes in gene expression regulation. To our knowledge no other detailed comparative studies on transcriptional response after exposure to alpha particles and electrons have been reported. Altogether, it is likely that the number of transcripts involved in radiation-induced response depends with varying degree on time after administration, dose-rate, and absorbed dose. Further research is necessary to fully elucidate the relationship between these parameters in the aspect of the radiation-induced response.

In the present study, 25 genes were recurrently regulated in all exposed groups. Generally, these genes were regulated in the same direction (either all genes were upregulated or downregulated), which suggests similar involvement of these genes in the response to different exposures. Several of the 25 recurrently regulated genes were previously identified in mouse thyroid tissue 24 h after ^211^At administration (0.05–32 Gy to thyroid) [11]. This finding suggests that the recurrently regulated genes play a role in how mouse thyroid tissue responds to ^211^At exposure.

Few of the 102 previously proposed genes were identified in the present study. This is in agreement with our previous studies on thyroid tissue after ^131^I and ^211^At administration and in kidneys after ^177^Lu-octreotate administration [11, 13, 16]. An explanation may be that most of these biomarkers were defined in *in vitro* studies and after acute external exposure. Thus, to identify biomarkers for internal radiation exposure in organisms, we clearly propose *in vivo* studies, ideally using human tissue. In the present study, the *Cdkn1a* (cyclin-dependent kinase inhibitor 1) and *Gadd45g* (growth arrest and DNA-damage-inducible protein) genes were regulated in three and four exposed groups, respectively. The gene products for *Cdkn1a* and *Gadd45g* are both involved in cellular responses to DNA damage and genotoxic stress and have been previously associated with radiation-induced response [28, 29]. Additionally, none of the previously proposed biomarkers were detected 168 h after ^211^At administration, suggesting that future studies should include a wider range of temporal end-points when searching for radiation-related biomarkers.

We identified genes exclusively regulated at either 1 or 6 h, after 1.4 Gy, or after 1.7 kBq administration. These genes may potentially be used to distinguish a response between different exposure conditions, and might be useful for triage situations, although the majority of the genes were regulated with small changes compared with non-irradiated controls. The presence of exposure-specific genes also suggests that the response to different exposure conditions results in regulation of different genes. Altogether, these data revealed: 1) no change in direction of regulation of genes exclusively regulated at either 1 or 6 h after administration even with changes in absorbed dose and dose-rate, 2) no change in direction of regulation of genes exclusively regulated after administration of 1.7 kBq, even though the absorbed dose and time after administration were distinctly different, and 3) a change in direction of regulation of genes exclusively regulated at 1.4 Gy but at different time-points and initial dose-rates. Additionally, genes regulated in the same direction also had similar changes in fold change (Additional File 1). Regulation of gene expression may vary over time; therefore, the mixed direction of regulation of genes exclusively regulated at 1.4 Gy may be expected. However, mixed direction of regulation was not seen for the 1.7 kBq administrations when time after administration increased. Conclusively, this suggests that the dose-rate may have a larger impact on direction of regulation of these exclusively regulated genes compared with absorbed dose and time after administration. Additionally, only one gene (*Casp1*) was (down)regulated at all time-points after 1.7 kBq administration. The gene product of *Casp1* is involved in the formation of the inflammasome [30].

According to enrichment of regulated genes, biological processes associated with immune response were prevalent in the radiation-induced response 1, 6, and 168 h after 1.7 kBq administration. However, immune response-related biological processes at 6 h were different compared with those identified at 1 and 168 h. These findings (together with few biological processes related to immune response at 1.4 Gy at 1 and 6 h) suggest that immune response may involve different phases at different time periods after administration of 1.7 kBq ^211^At (0.023, 0.32, 1.8 Gy). This is supported by data at 24 h after ^211^At administrations (0.05–32 Gy), where few biological processes related to immune response were identified [11]. Additionally, few immunological processes were identified at 1.4 Gy, 1 and 6 h after administration of ^211^At. This suggests that the radiation-induced response involved more immunological processes at low absorbed dose levels at 1 and 6 h. Few GO terms categorized as part of an inflammatory response were identified in the present study, which is in agreement with our previous data 24 h after ^211^At administration [11]. A review paper on radiation-induced biological effects describes that inflammatory and immunologic responses are orchestrated with an acute inflammatory response at early time-points followed by tissue remodeling [31]. It is likely that this scenario is different for internal radiation exposure with protracted exposure and decreasing dose-rate, and e.g. GO terms associated with inflammatory response were only identified at 168 h in the present work. Further studies are necessary to elucidate how inflammatory and immunological processes are regulated in response to differences in radiation quality, dose-rate, absorbed dose and time after administration of radionuclides.

Few GO terms related to DNA and gene expression integrity were identified in the present study. This is in agreement with previous results on transcriptional changes in thyroid tissue after ^131^I and ^211^At [11, 13]. Monte Carlo simulations have shown that 1.2 Gy after ^211^At irradiation corresponds to a mean value of one alpha particle track per thyroid follicular cell [32]. Assuming 10–100 DNA double strand breaks per Gy and that 10–15 DNA double strand breaks per cell are needed for initiation of the DNA repair machinery on the protein level, the absorbed dose level in the present study and in our previous studies may not be high enough to trigger *de novo* protein synthesis of molecules involved in DNA repair. Also, protracted irradiation results in fewer DNA double strand breaks per time unit compared with e.g. acute exposure.

Many GO terms related to metabolism were identified in the present study, especially for the highest dose-rate. Additionally, GO terms related to cellular integrity were identified in the present study, but different cellular integrity GO terms were detected in the different groups, indicating differences in the response to different exposure conditions.

## Conclusions

The present study contains a comprehensive assessment of how variations in exposure parameters affect transcriptional regulation. The regulation profiles were clearly dependent on both absorbed dose and time after exposure, but initial dose-rate was the most influential parameter. Functional annotation of regulated genes revealed exposure-specific effects on biological processes. Additionally, we identified several recurrently regulated genes that may be important in mouse thyroid tissue response to ^211^At exposure.

## Supporting Information

S1 Table Genes specific for certain exposure conditions. Genes regulated for the following exposure: 1 h or 6 h after administration, 1.4 Gy absorbed dose, or administration of 1.7 kBq ^211^At. FILE NAME: Supporting Table 1 (S1 Table). FILE TYPE: MICROSOFT EXCELSPREADSHEET,.xlsx. TITLE OF DATA: Genes specific for certain exposure conditions. DESCRIPTION OF DATA: Genes regulated for the following exposure: 1 h or 6 h after administration, 1.4 Gy absorbed dose, or administration of 1.7 kBq ^211^At.
(XLSX)
